# Therapeutic effect of *Echinococcus granulosus* cyst fluid on bacterial sepsis in mice

**DOI:** 10.1186/s13071-023-06021-7

**Published:** 2023-12-08

**Authors:** Shuying Wang, Donghui Jiang, Feifei Huang, Yayun Qian, Meitao Qi, Huihui Li, Xiaoli Wang, Zhi Wang, Kaigui Wang, Yin Wang, Pengfei Du, Bin Zhan, Rui Zhou, Liang Chu, Xiaodi Yang

**Affiliations:** 1 First Affiliated Hospital of Bengbu Medical College, Bengbu, 233000 China; 2 Anhui Key Laboratory of Infection and Immunity of Bengbu Medical College, Bengbu, 233000 China; 3 Department of Pediatrics, Anqing First People’s Hospital of Anhui Medical University, Anqing, 246000 China; 4 Department of Critical Care Medicine, Affiliated Hospital of Jiangnan University, Wuxi, 214122 China; 5 National School of Tropical Medicine, Baylor College of Medicine, Houston, TX 77030 USA; 6 Second Affiliated Hospital of Bengbu Medical College, Bengbu, 233000 China

**Keywords:** Sepsis, *Echinococcus granulosus*, Cyst fluid, Macrophage, Immunomodulation

## Abstract

**Background:**

The primary pathophysiological process of sepsis is to stimulate a massive release of inflammatory mediators to trigger systemic inflammatory response syndrome (SIRS), the major cause of multi-organ dysfunction and death. Like other helminths, *Echinococcus granulosus* induces host immunomodulation. We sought to determine whether *E. granulosus* cyst fluid (*Eg*CF) displays a therapeutic effect on sepsis-induced inflammation and tissue damage in a mouse model.

**Methods:**

The anti-inflammatory effects of *Eg*CF were determined by in vitro culture with bone marrow-derived macrophages (BMDMs) and in vivo treatment of BALB/C mice with cecal ligation and puncture (CLP)-induced sepsis. The macrophage phenotypes were determined by flow cytometry, and the levels of cytokines in cell supernatants or in sera of mice were measured (ELISA). The therapeutic effect of *Eg*CF on sepsis was evaluated by observing the survival rates of mice for 72 h after CLP, and the pathological injury to the liver, kidney, and lung was measured under a microscope. The expression of TLR-2/MyD88 in tissues was measured by western blot to determine whether TLR-2/MyD88 is involved in the sepsis-induced inflammatory signaling pathway.

**Results:**

In vitro culture with BMDMs showed that *Eg*CF promoted macrophage polarization to M2 type and inhibited lipopolysaccharide (LPS)-induced M1 macrophages. *Eg*CF treatment provided significant therapeutic effects on CLP-induced sepsis in mice, with increased survival rates and alleviation of tissue injury. The *Eg*CF conferred therapeutic efficacy was associated with upregulated anti-inflammatory cytokines (IL-10 and TGF-β) and reduced pro-inflammatory cytokines (TNF-α and INF-γ). Treatment with *Eg*CF induced Arg-1-expressed M2, and inhibited iNOS-expressed M1 macrophages. The expression of TLR-2 and MyD88 in *Eg*CF-treated mice was reduced.

**Conclusions:**

The results demonstrated that *Eg*CF confers a therapeutic effect on sepsis by inhibiting the production of pro-inflammatory cytokines and inducing regulatory cytokines. The anti-inflammatory effect of *Eg*CF is carried out possibly through inducing macrophage polarization from pro-inflammatory M1 to regulatory M2 phenotype to reduce excessive inflammation of sepsis and subsequent multi-organ damage. The role of *Eg*CF in regulating macrophage polarization may be achieved by inhibiting the TLR2/MyD88 signaling pathway.

**Graphical Abstract:**

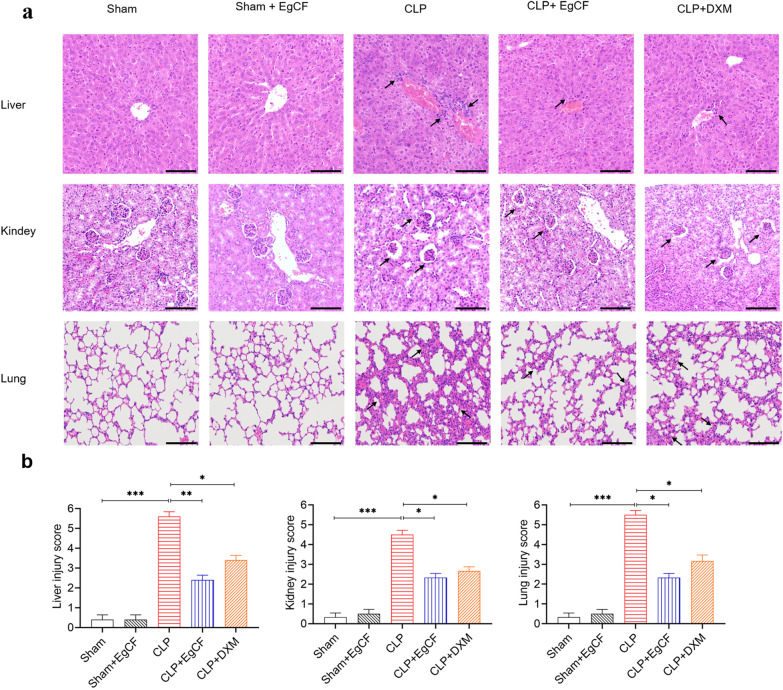

## Background

Sepsis is the overwhelming immune response to serious bacterial infection that can lead to tissue damage, organ failure, and death [1, 2]. It is a life-threatening condition and one of the most severe complications in patients with acute infections [3, 4]. A conservative estimate of 48.9 million cases of sepsis and about 11 million deaths from sepsis worldwide was reported as of 2017 [5]. Therefore, a better understanding of the pathogenesis of sepsis and the search for a better treatment have become essential for reducing its mortality. Recent studies have shown that the essence of sepsis is an imbalanced immune response between pro-inflammatory and anti-inflammatory mechanisms [6, 7]. In sepsis, when macrophages and other innate immune cells detect bacterial pathogens or are activated by bacterial endotoxin, they release many pro-inflammatory cytokines to produce inflammatory cytokine storms called systemic inflammatory response syndrome (SIRS) to eliminate and clear infectious organisms [8, 9]. The inflammation reactions damage the structure and function of vital organs such as the kidney, liver, and lung [10–13] and even lead to multiple organ dysfunction and failure. Simultaneously, some immune cells can be activated to unleash an anti-inflammatory response characterized by the release of anti-inflammatory cytokines. This is called compensatory anti-inflammatory response syndrome (CARS), and its purpose is to restore homeostasis and reduce immunopathological damage; however, it may cause immunosuppression with delayed pathogen clearing [14]. Therefore, balancing the inflammatory and anti-inflammatory responses has become the focus of treating sepsis.

Macrophages, as important intrinsic immune cells during inflammatory response, play a crucial role in the immune responses and become the central target cells in the treatment of sepsis [15–17]. The heterogeneity of macrophages leads to a diversity of functions. Activated macrophages can exhibit different phenotypes and exert different pro- or anti-inflammatory effects depending on the surrounding environmental signals [18]. M1-type macrophages are typically characterized by high levels of oxidative metabolites (reactive oxygen species and NO) and pro-inflammatory cytokines (tumor necrosis factor alpha [TNF-α] and interleukin [IL]-6), which amplify the inflammatory cytokine storm and aggravate sepsis [19]. In contrast, M2-type macrophages produce high levels of Arg-1, CD206, IL-10, and transforming growth factor beta (TGF-β), which play an immunomodulatory role and have a therapeutic effect on sepsis [20]. The ratio of M1-type to M2-type macrophages influences the regression of sepsis [21].

Toll-like receptors (TLRs), a family of receptors expressed on the membrane of innate immune cells associated with cell recognition [22, 23], are intermediate in the generation of a persistent systemic “inflammatory waterfall” and closely associated with the development of sepsis [24]. Upon pathogen recognition through TLR4 or TLR2, the MyD88 signal pathway is stimulated and mediates inflammatory immune responses [25] to clear the invader pathogens and induce immunopathology as well. Knockdown of MyD88 was found to reduce lipopolysaccharide (LPS)-induced lung injury and fibrosis [26]. Even though both TLR2 and TLR4 are major receptors of gram-negative bacteria-produced LPS [27], in our pre-experiment screening test, we only found that the TLR2/MyD88 signaling pathway was significantly induced by LPS. Uncontrolled TLR2/MyD88 signal activation may cause excessive release of inflammatory mediators, leading to severe inflammatory reactions and causing tissue damage [28]. Therefore, the TLR2/MyD88 signaling pathway can serve as an essential marker for the severity of sepsis and treatment efficacy.

In 1989, Strachan [29] proposed the “hygiene hypothesis” based on the observed correlation between cleanliness of helminth infection and increased autoimmune and allergic diseases. Further evidence has shown that helminths secrete a variety of proteins that play a role in modulating the host immune response to reduce immune attack on the parasites as a survival strategy while reducing the hypersensitivity status of the host immune system during some autoimmune diseases or inflammatory diseases. Based on the immunomodulatory functions of helminth infection or helminth-produced proteins, the helminth therapy or the worm-derived products of autoimmune or inflammatory diseases (e.g., excretory/secretory products [SPs]), extracellular vesicles [EVs], recombinant protein molecules) have been successfully applied to experimentally treat some autoimmune or inflammatory diseases [30–35]. The immunomodulation of helminth-derived proteins includes regulating the function of antigen-presenting cells, activating Treg cells to promote anti-inflammatory factors such as TGF-β and IL-10, and stimulating M2 macrophage polarization [36–40].

*Echinococcus granulosus* is a parasitic tapeworm found in humans and animals, and its larvae can survive in human tissue (liver or lung) as hydatid cysts for up to 53 years [41], possibly by releasing immunomodulatory proteins to activate host regulatory network elements. Animal experiments have confirmed the therapeutic effect of *E. granulosus* infection on intestinal inflammation and asthma [42, 43]. However, the application of live helminth infection is risky to human health and not acceptable [44, 45]. Instead, helminth-derived proteins can avoid the risk of helminth infection and produce similar anti-inflammatory effects [32]. Our previous study showed that antigen B secreted by *E. granulosus* could modulate host immune responses to inhibit Th17 and promote Treg differentiation, thus alleviating the airway inflammatory response to asthma [46]. In the present study, we sought to determine whether *E. granulosus* cyst fluid (*Eg*CF) is able to exert a similar immunomodulatory effect on sepsis and whether macrophages are involved in the anti-inflammatory differentiation during treatment of sepsis.

## Methods

### Preparation of *Eg*CF

Fertile hydatid cysts were surgically removed from an infected patient in Xingjiang, China. The patient signed informed consent form. The *Eg*CF was collected by centrifuging the fluid collected from the cysts at 12,000×*g* for 30 min at 4 °C to remove protoscolices and other worm materials. The potentially contaminated endotoxin in *Eg*CF was removed using a ToxOut™ High Capacity Endotoxin Removal Kit (BioVision, Palo Alto, CA, USA), and the residual endotoxin was assessed using a ToxinSensor™ Chromogenic Limulus Amebocyte Lysate (LAL) Endotoxin Assay Kit (GenScript Biotechnology, Nanjing, China). The protein concentration in the collected *Eg*CF was determined using a bicinchoninic acid (BCA) protein assay kit (Biosharp, Hefei, China).

### Animals

Specific-pathogen-free male BALB/c mice, 6–8 weeks old, were obtained from Custer Laboratory Animal Center, Suzhou. All animal experiments were carried out in accordance with the regulations for the administration of Affairs Concerning Experimental Animals in China using the approved protocol (approval no. LAEC-2022-413).

### Preparation and induction of mouse bone marrow-derived macrophages (BMDMs)

Donor mice were sacrificed by cervical dislocation, and the femur and tibia were recovered and washed twice with PBS. The bone marrow cavities were rinsed out with an incomplete Dulbecco's modified Eagle medium (DMEM; Thermo Fisher, MA, USA), and the rinse solution was filtered with a 200-mesh screen to remove fragments. The cells in the rinse solution were spun down and resuspended in a complete DMEM medium containing 10% fetal bovine serum (FBS) and 1× penicillin (100 U/ml)/streptomycin (100 μg/ml). The cells were cultured in complete DMEM supplemented with 20 ng/ml mouse macrophage colony-stimulating factor (M-CSF) (R&D Systems, MN, USA) for 7 days. The mature BMDMs were confirmed using flow cytometry with markers of CD11b^+^F4/80^+^.

### Effect of *Eg*CF on macrophage polarization in vitro

The mature BMDMs collected from the above culture were divided into five groups: (i) incubated with DMEM only (DMEM group), (ii) incubated with LPS (100 ng/ml) (LPS group), (iii) incubated with IL-4 (20 ng/ml) (IL-4 group), (iv) co-incubated with LPS (100 ng/ml) and *Eg*CF (1 ug/ml) (*Eg*CF + LPS group), and (v) incubated with *Eg*CF only (1 ug/ml) (*Eg*CF group). After being cultured for 24 h, the cells were collected for measuring CD86 and CD206 expression on the surface using flow cytometry, and the culture supernatant was collected for measuring the levels of TNF-α, interferon gamma (IFN-γ), TGF-β, and IL-10 using an enzyme-linked immunosorbent assay (ELISA) kit with specific antibodies (Dakewe Biotech, Beijing, China).

### Analysis of macrophage polarization by flow cytometry

The collected BMDM cells were first treated with Fixable Viability Dye eFluor 510 (BioLegend, Inc., San Diego, CA, USA) for 10 min to differentiate live/dead cells. After being blocked with Fc receptor blocker (BioLegend, Inc., USA) for 10 min, the cells were incubated with FITC-anti-F4/80, BV605-anti-CD11b, and APC-anti-CD86 antibodies (BioLegend, Inc., USA) for surface marker staining for 25 min. For staining CD206, the cells were fixed and permeabilized using a Thermo fixation/permeabilization kit (Thermo Fisher Scientific, USA), then stained with PE-anti-CD206 (BioLegend, Inc., USA) for 30 min. The flow cytometry for these staining cells was performed using a DxP Athena™ flow cytometer (Cytek Biosciences Inc., CA, USA). The isotype-matched immunoglobulins (BioLegend, Inc., USA) and fluorescence minus one (FMO) were used as controls for non-specific staining as a baseline.

### Mouse model of sepsis induced by cecal ligation and puncture (CLP)

Based on previous publications, a clinically relevant septic rodent model was established by CLP as described previously [47]. Briefly, mice were fasted for 12 h with only access to water, then anesthetized under isoflurane inhalation. The abdominal cavity was opened and the cecum was tightly ligated with a 3.0 silk suture at 1.0 cm from the apex. The puncture was made with an 18-gauge needle to squeeze out a small amount of stool and gently return the punctured cecum to the abdomen. The opened abdominal cavity was sutured layer by layer. In the control group, only laparotomy was performed without ligation and perforation. The general condition and survival rate of mice were observed for up to 72 h.

### Treatment of sepsis with *Eg*CF

Eighty mice were randomly divided into five groups with 16 mice in each group. The first three groups of mice were treated with CLP; 30 min after the surgery, each group of 16 mice was treated intraperitoneally with 1 ug *Eg*CF (CLP + *Eg*CF), 0.3 mg/kg dexamethasone (CLP + DXM) or PBS only as control (CLP) in a total volume of 100 μl, respectively. As normal controls, the remaining two groups of mice were treated with the sham operation, and then treated with the same dose of *Eg*CF (sham + *Eg*CF) or PBS (sham). Six mice from each group were sacrificed 12 h after treatment, the blood was obtained to separate sera for measuring the levels of different cytokines, and the liver, kidney, and lung were collected for histopathological examination. The remaining 10 mice from each group were observed for 72 h to determine the survival rate.

### Serological test for cytokine profile and biochemical markers of organ damage

Serological levels of alanine aminotransferase (ALT) and aspartate aminotransferase (AST) were measured to determine liver injury, and the levels of blood urea nitrogen (BUN) and creatinine (Cr) were measured to assess kidney injury induced by sepsis. The serological levels of pro-inflammatory cytokines (TNF-α, IFN-γ) and regulatory anti-inflammatory cytokines (IL-10, TGF-β) were detected by using the same ELISA kit mentioned above.

### Histopathological changes in tissues of the liver, kidney, and lung

Liver, kidney, and lung were collected from six mice in each experimental group after being euthanized 12 h after treatment, and fixed in 4% paraformaldehyde. The tissue was cut into 5-mm sections and stained with hematoxylin–eosin (HE). The histological liver injury was determined according to the degree of liver cell cytoplasmic vacuolation, hepatic sinus congestion, and liver cell necrosis [48], and corresponding scores were evaluated based on the criteria listed in Table 1. Histological renal injury was assessed based on the degree of damaged tubules and atrophic glomerulus score [49], as shown in Table 2. Histological lung injury scores were determined based on alveolar hyperemia, alveolar edema, neutrophil filtration, and alveolar septum thickening [50], as shown in Table 3.

**Table 1:** Liver injury score parameters

Hepatocellular edema, congestion, cell immersion	Injury scores
No	0 (normal)
< 25%	1 (moderate)
25–50%	2 (severe)
50–70%	3 (extremely severe)
> 75%	4 (critical)

**Table 2:** Kidney injury score parameters

Kidney tube injury and glomerular reduction	Injury scores
No	0 (normal)
< 25%	1 (moderate)
25–50%	2 (severe)
50–70%	3 (extremely severe)
> 75%	4 (critical)

**Table 3:** Lung injury score parameters

Alveolar congestion, alveolar wall thickness, and cell infiltration	Injury scores
No	0 (normal)
< 25%	1 (moderate)
25–50%	2 (severe)
50–70%	3 (extremely severe)
˃ 75%	4 (critical)

### Real-time quantitative polymerase chain reaction (qPCR)

Total RNA of the liver, kidney, and lung tissues was extracted using TRIzol Up (TransGen Biotech, China) and reverse-transcribed to complementary DNA (cDNA) using the EasyScript^®^One-Step gDNA Removal and cDNA (TransGen Biotech, China). The relative messenger RNA (mRNA) expression of macrophage polarization-related markers inducible nitric oxide synthase (iNOS; M1) and Arg-1 (M2) in tissues of the liver, kidney, and lung were determined using PerfectStart^®^ Green qPCR SuperMix (TransGen Biotech, China) on a Roche LightCycler^®^ 96 real-time PCR system (Roche Molecular Systems, Inc., Pleasanton, CA, USA). The relative mRNA expression was calculated by the formula 2^−△△Cq^ compared with housekeeper gene GAPDH. The primers for molecules mentioned above were synthesized by Sangon Biotech (Shanghai, China).

### Expression of TLR2 and MyD88 in liver, kidney, and lung tissues of mice by western blotting

The total protein of the liver, kidney, and lung was extracted and the concentration was measured using BCA (Biosharp, Hefei, China). The tissue samples were separated by sodium dodecyl sulfate–polyacrylamide gel electrophoresis (SDS-PAGE) and transferred to a polyvinylidene fluoride (PVDF) membrane. The membrane was blocked with 5% skim milk for 2 h, then recognized by rabbit anti-MyD88 antibody (1:4000), rabbit anti-TLR2 antibody (1: 3000), or rabbit anti-β-actin antibody (1:5000) (all from Cell Signaling Technology, Danvers, MA, USA) overnight at 4 °C. Sheep anti-rabbit IgG-HRP (1:7000) (Biosharp, Heifei, China) was used as secondary antibody. Immunoreactive protein bands were visualized by enhanced chemiluminescence (Bio-Rad, Hercules, CA, USA). The relative expression levels of MyD88 and TLR-2 were determined by the ratio of the identified density to the β-actin density of the control.

### Statistical analysis

Statistical analysis was performed using one-way analysis of variance (ANOVA) for multi-group comparisons using unpaired, two-tailed Student’s *t*-test with Bonferroni adjustment, or ANOVA for multiple comparisons using GraphPad Prism 6.0 software (GraphPad Software, Inc., La Jolla, CA, USA). All data were presented as the mean ± standard error of the mean (SEM). Kaplan–Meier survival analysis was used to compare the difference in survival rates among groups. *P* < 0.05 was considered statistically significant.

## Results

### Protein components of *Eg*CF

**Fig. 1:**
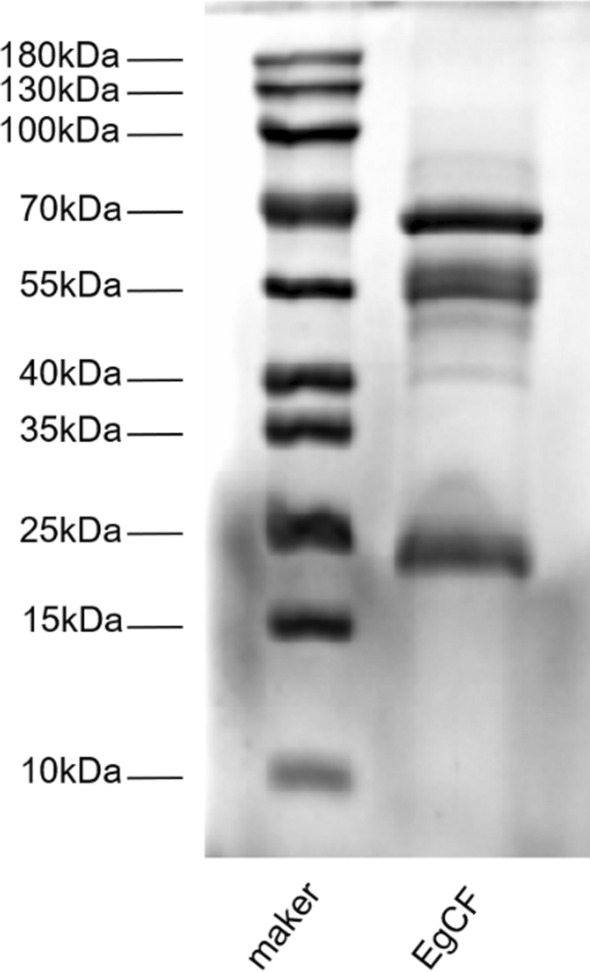
SDS-PAGE analysis of *Eg*CF. Total 40 μg of *Eg*CF was separated by 12% polyacrylamide gel electrophoresis and stained with Coomassie blue

After separation by SDS-PAGE, *Eg*CF appeared as several major bands at approximately 25 kDa, 55 kDa, and 70 kDa. (Fig. 1).

### *Eg*CF promotes M2 macrophage polarization and inhibits LPS-induced M1 macrophages

**Fig. 2:**
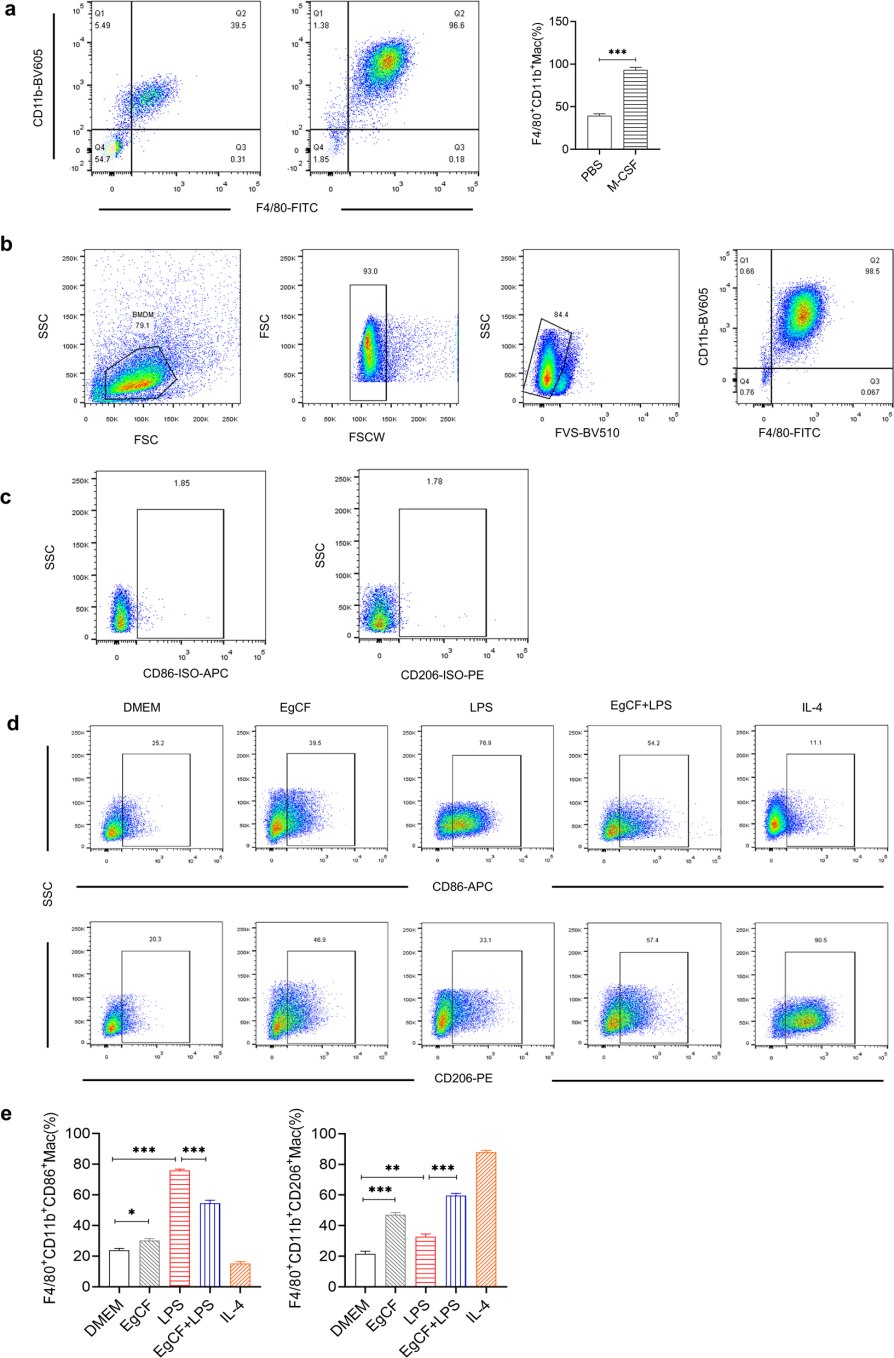
*Eg*CF stimulated BMDM differentiation to M2 macrophages. **a** The mature BMDMs were obtained by incubating mouse bone marrow cells (adherent) with M-CSF for 7 days, defined as CD11b^+^F4/80^+^ subpopulations using FACS. **b** Gating strategy on flow cytometry to differentiate dead cells and adhere cells and BMDMs labeled with CD11b+F4/80+. **c** The isotype-matched immunoglobulins were used as a control for non-specific staining as a baseline. **d**, **e** *Eg*CF enhanced M2 macrophages under LPS-stimulating conditions. BMDMs were incubated with *Eg*CF (1 ug/ml), LPS (100 ng/ml), IL-4 (20 ng/ml), *Eg*CF + LPS, or DMEM, respectively, for 24 h. The M1 (CD86) and M2 (CD206) markers were detected using FACS. The results were shown as the mean ± SEM for each group (*n* = 6). *P < 0.05, **P < 0.01, ***P < 0.001

Bone marrow cells were induced with M-CSF for 7 days, and maturity of BMDMs was confirmed by flow cytometry with F4/80^+^ and CD11b^+^ (Fig. 2a). More than 99.6% of BMDMs were F4/80^+^CD11b^+^, which was significantly higher than the group incubated with PBS only (*t*-test: *t*_(8)_ = 32.62, *P* < 0.001).

To investigate the effect of *Eg*CF on macrophage polarization, the mature BMDMs were incubated with *Eg*CF for 24 h and CD86 (M1) and CD206 (M2) were measured (Fig. 2b). The flow cytometry results showed that *Eg*CF significantly induced F4/80^+^CD11b^+^CD206^+^ (M2) macrophages while it slightly induced (M1) macrophages (Fig. 2b). As positive controls, LPS strongly stimulated F4/80^+^CD11b^+^CD86^+^ macrophages (M1) and IL-4 stimulated F4/80^+^CD11b^+^CD206^+^ macrophages (M2) relative to DMEM plain medium control (ANOVA: *F*_(4, 25)_ = 278.39, *P* < 0.0001 for the former and F_(4, 25)_ = 278.39, *P* < 0.0001 for the latter) (Fig. 2d, e). Interestingly, under continuing stimulation of LPS, a similar situation as sepsis condition, *Eg*CF still has the ability to reduce M1 (F4/80^+^CD11b^+^CD86^+^) and boost M2 (F4/80^+^CD11b^+^CD206^+^) macrophages (Fig. 2d, e).

### *Eg*CF inhibits pro-inflammatory cytokines and stimulated regulatory cytokines secreted by LPS-stimulated macrophages

**Fig. 3:**
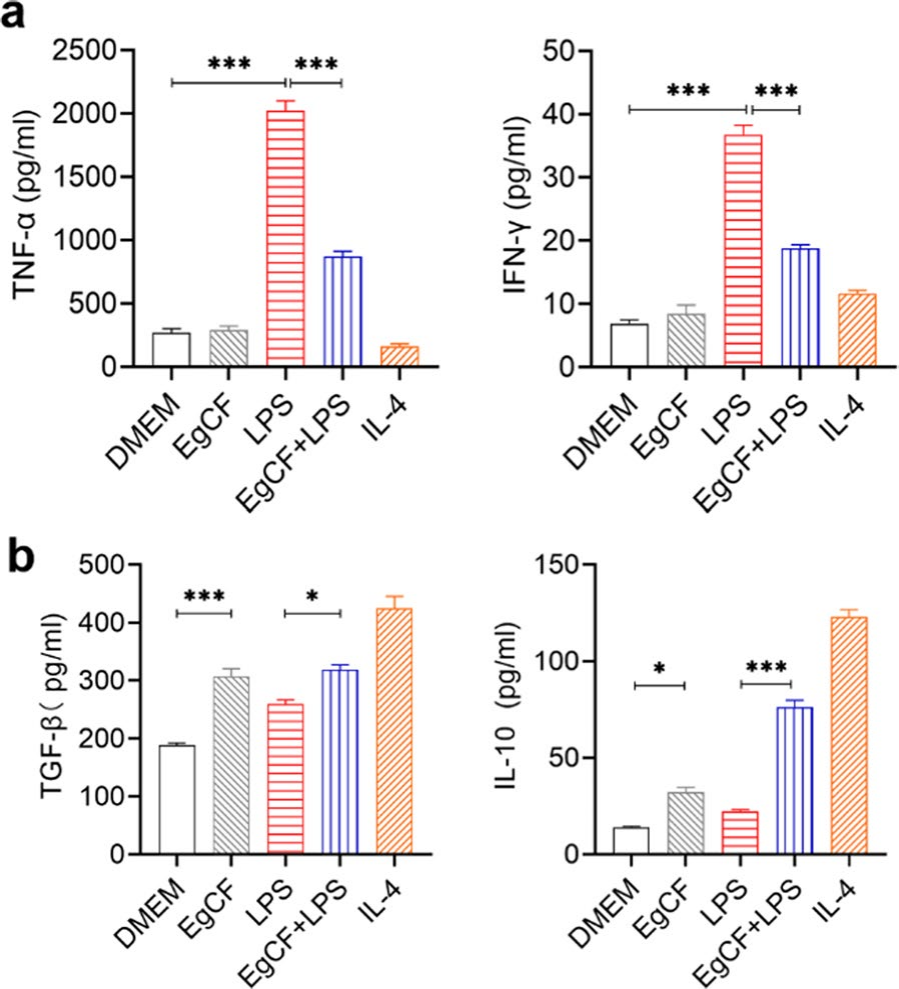
*Eg*CF stimulated M2 type macrophage with regulatory cytokine secretions and inhabited LPS-induced M1 type cytokines. Mature BMDMs were incubated with *Eg*CF (1 ug/ml), LPS (100 ng/ml), IL-4 (20 ng/ml), or *Eg*CF + LPS for 24 h. **a** The levels of M1-related cytokines TNF-α and IFN-γ were measured in the supernatant by ELISA. **b** The levels of M2-related cytokines IL-10 and TGF-β were measured in the supernatant by ELISA. *n* = 6. The results are presented as mean ± SEM, *P < 0.05, ***P < 0.001

The levels of different types of cytokines in the BMDM culture supernatant were measured by enzyme-linked immunosorbent assay (ELISA). The results showed that LPS-stimulated macrophages secreted high levels of pro-inflammatory cytokines TNF-α and IFN-γ relative to the culture with plain DMEM medium (ANOVA: *F*_(4, 24)_ = 290.00, *P* < 0.0001; *F*_(4, 20)_ = 150.76, *P* < 0.0001, respectively); however, co-incubation with *Eg*CF significantly reduced LPS-stimulated TNF-α and IFN-γ levels (ANOVA: *F*_(4, 24)_ = 290.00, *P* < 0.0001; *F*_(4, 20)_ = 150.76, *P* < 0.0001, respectively) (Fig. 3a). On the other hand, *Eg*CF significantly stimulated BMDMs to secrete higher levels of regulatory cytokines TGF-β and IL-10 compared with the DMEM group (ANOVA: *F*_(4, 25)_ = 51.60, *P* < 0.0001; *F*_(4, 25)_ = 309.31, *P* < 0.0001, respectively). The higher levels of TGF-β and IL-10 were also observed in *Eg*CF + LPS group compared with the LPS group (ANOVA: *F*_(4, 25)_ = 51.60, *P* < 0.0001; *F*_(4, 25)_ = 309.31, *P* < 0.0001, respectively) (Fig. 3b). These cytokine profile data further demonstrate that *Eg*CF promotes macrophage polarization toward M2 type and inhibits LPS-induced M1 type macrophages.

### *Eg*CF improves the survival rate of septic mice

**Fig. 4:**
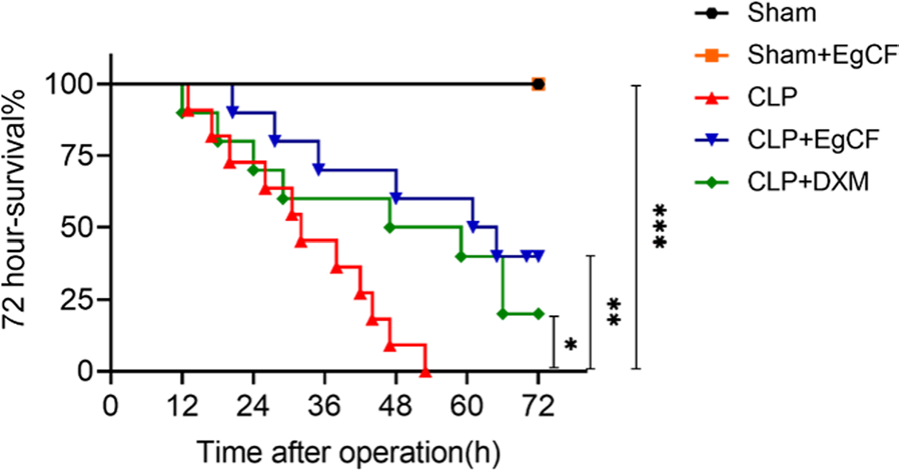
*Eg*CF treatment improved the survival rate of mice with CLP-induced sepsis. After CLP or sham operation, mice were injected intraperitoneally with *Eg*CF, DXM, or PBS. The survival rate was determined using Kaplan Meier method and compared by log-rank test. *n* = 10. *P < 0.05, **P < 0.01, ***P < 0.001

After CLP surgery, the 72-h survival status of septic mice was observed. It was found that all mice with CLP surgery without treatment died within 52 h. However, 40% of CLP mice survived for up to 72 h in the *Eg*CF-treated group (Kaplan–Meier analysis: *χ*^2^ = 8.48, *df* = 1, *P* < 0.0036) which is even higher than those CLP mice treated with corticosteroid DXM (Kaplan–Meier analysis: *χ*^2^ = 4.25, *df* = 1, *P* < 0.0393) (Fig. 4). All mice with sham surgery with or without *Eg*CF treatment survived for 72 h.

### *Eg*CF significantly improves pathological outcomes in septic mice

**Fig. 5:**
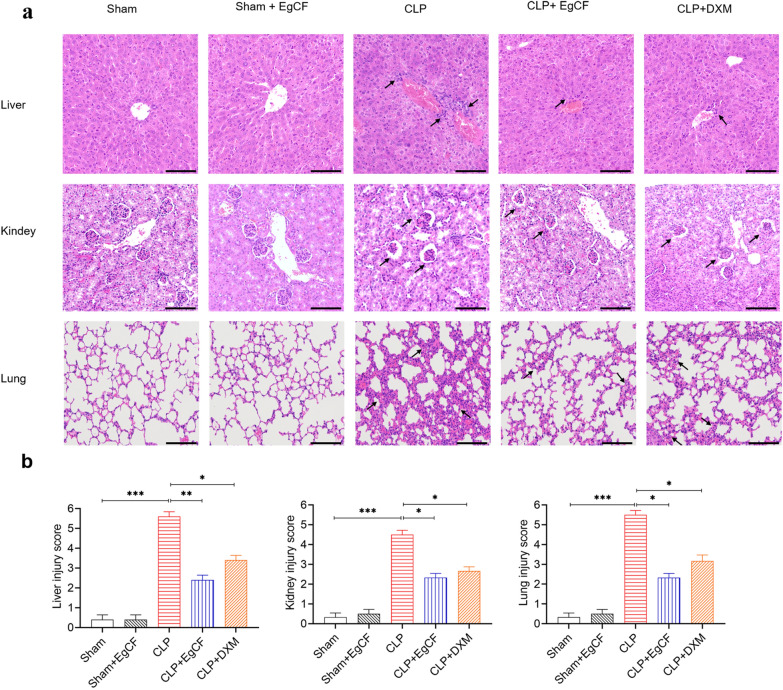
*Eg*CF reduced liver, kidney, and lung injury caused by CLP-induced sepsis. **a** The histopathology results of liver, kidney, and lung stained with H&E. The tissue was obtained from mice 12 h after CLP and treated with *Eg*CF, DXM, or PBS (arrows indicate inflammatory cell, shrunk glomerulus, and thickened interalveolar septum). **b** The pathological score comparison among different treated groups in liver, kidney, and lung tissue. The magnification × 200, scale bar = 100 µm. *n* = 6/group. The results are presented as mean ± SEM. *P < 0.05, **P < 0.01, ***P < 0.001

Liver tissue pathology (Fig. 5a, b): HE staining of liver tissue sections revealed that the group of mice with sham surgery had normal liver lobule structure, large hepatocyte nuclei in a radial arrangement, and no inflammatory cell infiltration. The CLP group mice lost typical liver lobule structures infiltrated with numerous inflammatory cells. Hepatocytes were obviously edematous and disordered arrangement, and the liver pathological damage score was significantly higher compared with the sham group (ANOVA: *F*_(4, 20)_ = 80.13, *P* < 0.0001). Hepatocyte edema, vacuolation, and inflammatory cell infiltration were significantly reduced in the CLP + *Eg*CF and CLP + DXM groups relative to the CLP group without treatment, with significantly reduced injury scores (ANOVA: *F*_(4, 20)_ = 80.13, *P* < 0.0001).

Kidney tissue pathology (Fig. 5a, b): The glomerulus structure of mice with CLP-induced sepsis was significantly shrunk and distorted, the renal tubule cell structure was damaged with renal pathological injury score significantly increased relative to the sham surgery group (ANOVA: *F*_(4, 25)_ = 63.27, *P* < 0.0001). After being treated with *Eg*CF, renal tissue damage was improved with reduced infiltration of inflammatory cells relative to mice without treatment (ANOVA: *F*_(4, 25)_ = 63.27, *P* < 0.0001). Mice treated with DXM showed a similar improvement in renal pathology as the *Eg*CF group.

Lung tissue pathology: (Fig. 5a, b): CLP-induced sepsis caused serious pathology in lung tissue with disordered alveolar structure and severe congestion and infiltration of inflammatory cells. The alveolar septum was significantly thickened. The lung pathological injury score was increased relative to the sham surgery group (ANOVA: *F*_(4, 25)_ = 79.75, *P* < 0.0001). After being treated with *Eg*CF, the lung pathological score was significantly improved as the group treated with DXM.

The above results showed that the *Eg*CF protein significantly improved the pathology of septic mice, reduced tissue damage, and promoted tissue repair.

### *Eg*CF significantly improves liver and kidney function in mice

**Fig. 6:**
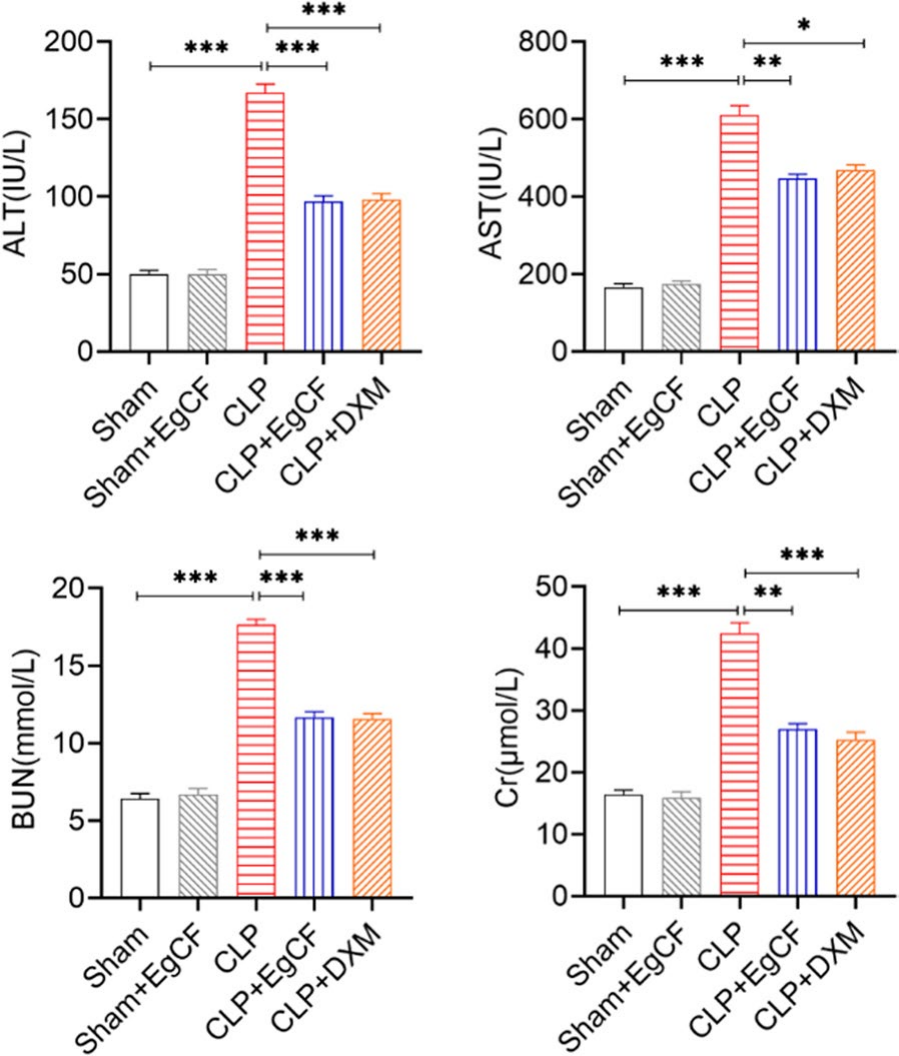
The levels of ALT, AST, BUN, and Cr were reduced in the sera of mice treated with *Eg*CF. The levels of ALT, AST, BUN, and Cr were measured in the sera from mice 12 h after CLP receiving different intervention. *n* = 6/group. The results are presented as mean ± SEM. *P < 0.05, **P < 0.01, ***P < 0.001, ****P < 0.0001

The serum levels of ALT, AST, the biomarkers for liver damage, and BUN and Cr, the biomarkers for kidney damage, were measured with a fully automated biochemical instrument. The results showed that the serum levels of ALT, AST, BUN, and Cr in mice of the CLP group were significantly higher than those in the sham group (ANOVA: *F*_(4, 25)_ = 158.63, *P* < 0.0001; *F*_(4, 25)_ = 186.20, *P* < 0.0001; *F*_(4, 25)_ = 173.26, *P* < 0.0001; *F*_(4, 25)_ = 91.40, *P* < 0.0001, respectively), indicating serious damage to liver and kidney functions. However, after being treated with *Eg*CF or DXM, the levels of ALT, AST, BUN, and Cr were significantly reduced relative to the group without treatment (ANOVA: *F*_(4, 25)_ = 158.63, *P* < 0.0001; *F*_(4, 25)_ = 186.20, *P* < 0.0001; *F*_(4, 25)_ = 173.26, *P* < 0.0001; *F*_(4, 25)_ = 91.40, *P* < 0.0001, respectively) (Fig. 6). These results indicate that *E. granulosus* cyst fluid proteins confer therapeutic effects on sepsis and improve liver and kidney functions.

### *Eg*CF reduces inflammatory cytokines and stimulates regulatory cytokines in septic mice

**Fig. 7:**
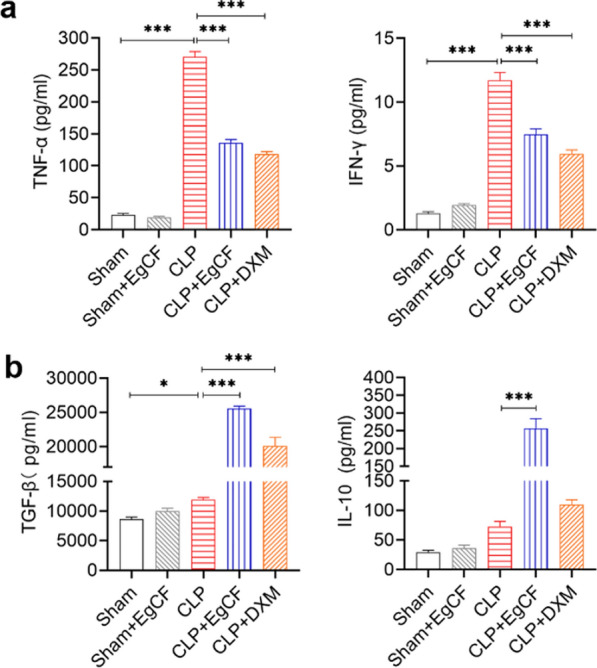
*Eg*CF reduced the inflammatory cytokines (TNF-α, IFN-γ) (**a**) and induced regulatory cytokines IL-10 and TGF-β (**b**) in sera of mice with CLP-induced sepsis. The cytokine levels were measured by ELISA. *n* = 6/group. The results are presented as mean ± SEM. *P < 0.05, **P < 0.01, ***P < 0.001

The levels of inflammatory cytokines TNF-α and IFN-γ, and regulatory cytokines TGF-β and IL-10 were measured in sera of mice with different treatment. The results showed that compared with the sham group, the serological levels of inflammatory cytokines TNF-α and IFN-γ were significantly induced in mice after CLP surgery (ANOVA: *F*_(4, 25)_ = 450.64, *P* < 0.0001; *F*_(4, 25)_ = 137.36, *P* < 0.0001, respectively), while the levels of regulatory cytokines TGF-β and IL-10 have no significant change. After being treated with TgCF, the levels of TNF-α and IFN-γ were significantly decreased compared with the CLP group without treatment (ANOVA: *F*_(4, 25)_ = 450.64, *P* < 0.0001), comparable to the DXM-treated group (*F*_(4, 25)_ = 137.36, *P* < 0.0001) (Fig. 7a). However, the serological levels of regulatory cytokines TGF-β and IL-10 were significantly stimulated after being treated with *Eg*CF compared with the group without treatment (ANOVA: *F*_(4, 25)_ = 126.49, *P* < 0.0001; *F*_(4, 25)_ = 44.30, *P* < 0.0001, respectively). Treatment with DXM also significantly boosted TGF-β secretion in sera (ANOVA: *F*_(4, 25)_ = 126.49, *P* < 0.0001; *F*_(4, 25)_ = 44.30, *P* < 0.0001, respectively), but the increase of IL-10 was not significant (Fig. 7b).

### *Eg*CF regulates macrophage polarization in liver, kidney, and lung tissues of septic mice

**Fig. 8:**
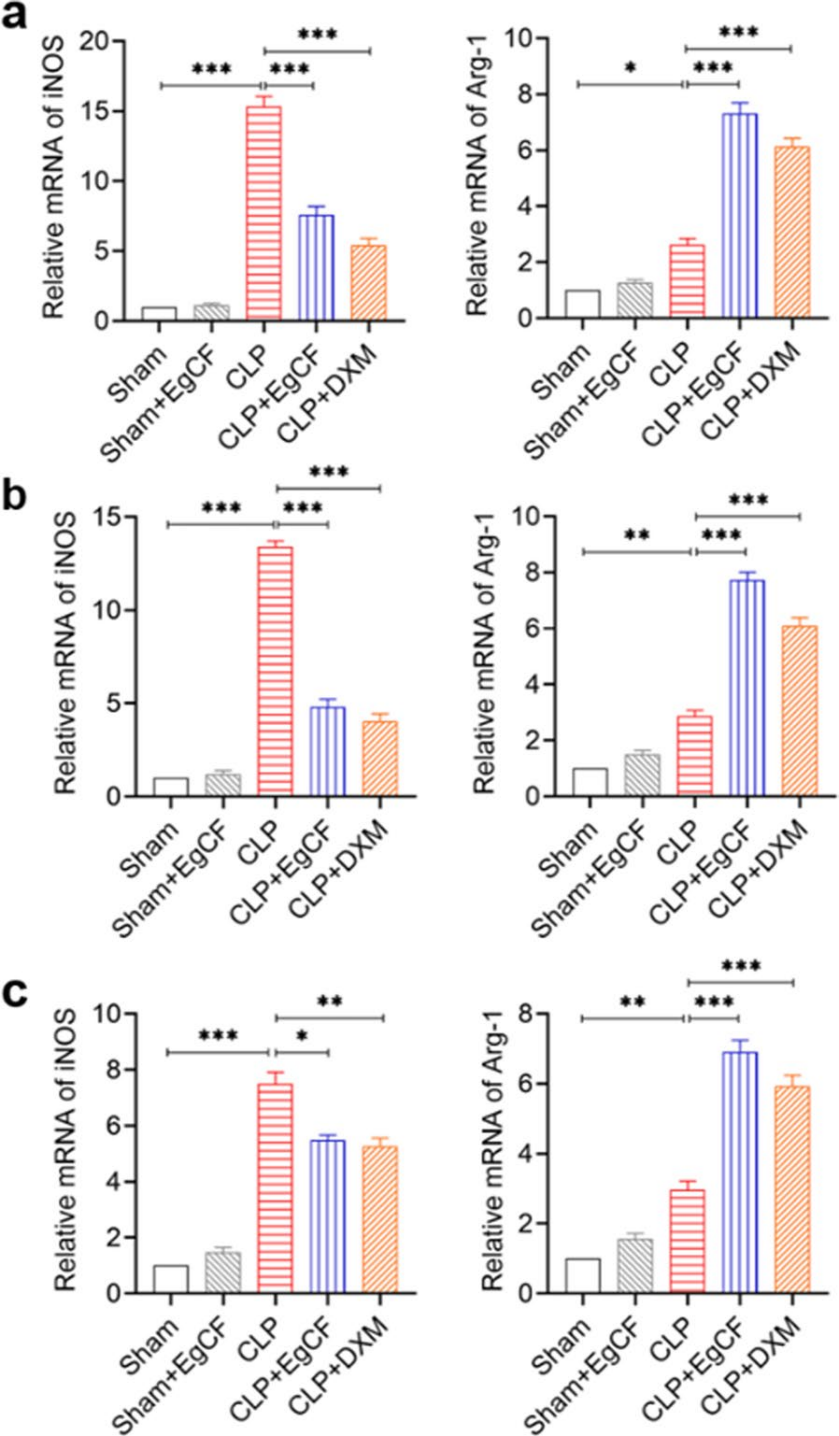
Treatment with *Eg*CF inhibited iNOS mRNA expression and stimulated Arg-1 mRNA expression in the liver (**a**), kidney (**b**), and lung tissues (**c**) measured by quantitative PCR compared with housekeeper GAPDH. *n* = 6/group. The results are presented as mean ± SEM. *P < 0.05, **P < 0.01, ***P < 0.001

To further investigate whether *Eg*CF protects septic mice by regulating macrophage polarization, RT-PCR was used to measure the expression levels of iNOS and Arg-1 mRNA in the liver, kidney, and lung tissues. The results showed that the transcriptional levels of iNOS (M1) were significantly increased in the liver, kidney, and lung tissue in CLP group mice relative to the sham group (ANOVA: *F*_(4, 25)_ = 149.83, *P* < 0.0001; *F*_(4, 25)_ = 280.13, *P* < 0.0001; *F*_(4, 25)_ = 127.24, *P* < 0.0001; respectively). The Arg-1 (M2) mRNA expression level was also increased (ANOVA: *F*_(4, 25)_ = 138.74, *P* < 0.0001, *F*_(4, 25)_ = 198.50, *P* < 0.0001; *F*_(4, 25)_ = 121.56, *P* < 0.0001, respectively) (Fig. 8a–c), indicating the inflammation caused by CLP-induced sepsis stimulated both M1 and M2 macrophages in these critical organs.

However, after being treated with *Eg*CF, the sepsis-induced iNOS mRNA expression was significantly inhibited in the liver (ANOVA: *F*_(4, 25)_ = 149.83, *P* < 0.0001), in kidney (*F*_(4, 25)_ = 280.13, *P* < 0.0001), and in lung tissue (F_(4, 25)_ = 127.24, *P* < 0.0001) compared with the CLP group without treatment. On the other hand, treatment with *Eg*CF significantly stimulated Arg-1 mRNA expression in liver (ANOVA: *F*_(4, 25)_ = 138.74, *P* < 0.0001), in kidney (*F*_(4, 25)_ = 198.50, *P* < 0.0001) and in lung tissue (*F*_(4, 25)_ = 121.56, *P* < 0.0001) compared with the group without treatment (Fig. 8a–c). Treatment with DXM also decreased the mRNA expression of iNOS and boosted the mRNA expression of Agr-1, but at a lower level compared with *Eg*CF. The results indicate that *Eg*CF inhibited M1 and stimulated M2 macrophages in critical organs of mice with CLP-induced sepsis.

### *Eg*CF inhibits the expression of TLR2 and MyD88 in liver, kidney, and lung tissues of septic mice

**Fig. 9:**
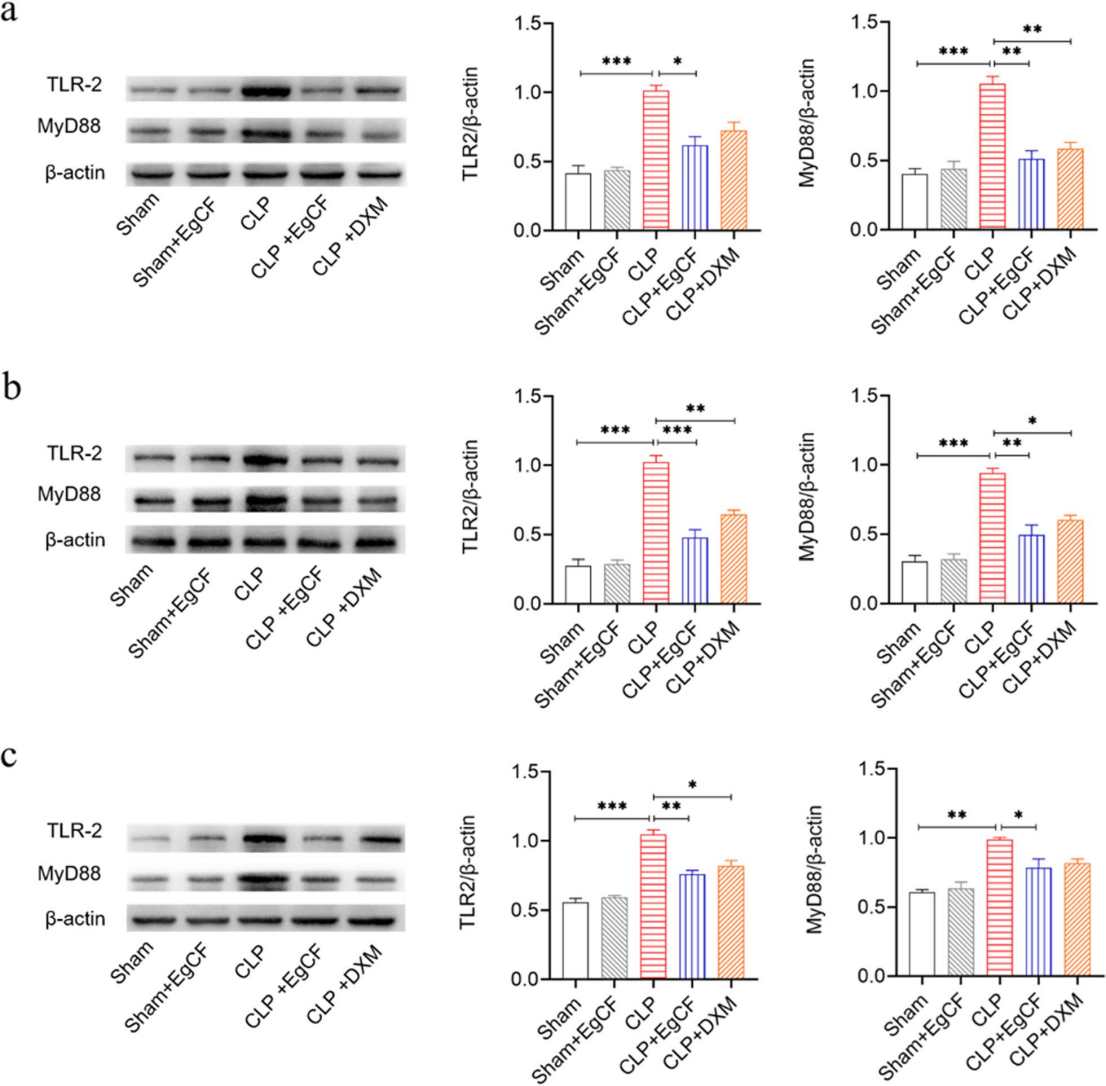
*Eg*CF suppressed the expression of TLR-2 and MyD88 in the liver (**a**), kidney (**b**), and lung (**c**) of mice with CLP-induced sepsis detected by western blot with specific antibody. The β-actin was used as a control. The density ratio of TLR-2/β-actin and MyD88/β-actin are shown on the right. The results are shown as the density mean ± SEM for each group. *n* = 3/group. The results are presented as mean ± SEM. *P < 0.05, **P < 0.01, ***P < 0.001

To verify whether the TLR2/MyD88 signaling pathway is involved in the therapeutic effect of *Eg*CF in septic inflammation, the expression of TLR2 and MyD88 proteins in the liver, kidney, and lung tissue of mice was measured using western blot with specific antibodies. The results showed that TLR2 and MyD88 levels were significantly increased in the liver tissue of septic mice after CLP surgery compared with the sham group (ANOVA: *F*_(4, 10)_ = 23.56, *P* < 0.0001; *F*_(4, 10)_ = 26.47, *P* < 0.0001, respectively). The increased levels of TLR2 and MyD88 were also observed in kidney tissue (ANOVA: *F*_(4, 10)_ = 50.64, *P* < 0.0001; *F*_(4, 10)_ = 31.66, *P* < 0.0001, respectively) and in lung tissue (ANOVA: *F*_(4, 10)_ = 44.01, *P* < 0.0001; *F*_(4, 10)_ = 14.69, *P* < 0.0001, respectively) after CLP surgery (Fig. 9a–c), indicating that TLR2 and MyD88 are involved in the inflammation of sepsis in CLP mice. Treatment with *Eg*CF significantly reduced the expression of TLR2 and MyD88 in the liver (ANOVA: *F*_(4, 10)_ = 23.56, *P* < 0.0001; *F*_(4, 10)_ = 26.47, *P* < 0.0001, respectively), kidney (*F*_(4, 10)_ = 50.64, *P* < 0.0001; *F*_(4, 10)_ = 31.66, *P* < 0.0001, respectively) and lung (*F*_(4, 10)_ = 44.01, *P* < 0.0001; *F*_(4, 10)_ = 14.69, *P* < 0.0001, respectively) of mice with CLP-induced sepsis relative to the septic mice without treatment (Fig. 9a–c). Treatment with DXM also reduced the expression of TLR2 and MyD88 in these tissues. The results indicated that the anti-inflammatory effect of *Eg*CF on serious bacterial infections such as sepsis may take place through inhibition of the TLR2 and MyD88 inflammatory signaling pathway.

## Discussion

Despite the availability of appropriate antibiotic therapy and supportive care, sepsis remains the leading cause of death in patients in intensive care unit [51]. The pathogenesis of sepsis involves multifaceted interactions between the infecting microorganism and the host. Effective control of the excessive immune responses at the early stage of sepsis is crucial for patients to survive the extreme inflammation condition [52, 53].

In recent years, helminths and their derivatives have been applied to treat autoimmune or inflammatory diseases experimentally or clinically by taking advantage of their modulatory functions on host immune responses. This immunomodulation includes the induction of Th2-biased response, activation of Treg cells, or alternatively activated macrophages to avoid hyper-immune responses to the exogenous and endogenous antigens or pathogens [54–56]. Our previous study has demonstrated that cystatin derived from helminth *Schistosoma japonicum* exerted immunomodulatory effects by inhibiting pro-inflammatory cytokines and promoting regulatory cytokines IL-10 and TGF-β and improved the survival rate of sepsis in a mouse model [48]. The ES products from adult *Trichinella spiralis* exerted a similar therapeutic effect on sepsis and protected sepsis-caused lung injury [25]. *Echinococcus granulosus* infection regulates host immune responses by inducing various immune cells to express regulatory phenotypes to reshape the immune homeostasis in the host [57, 58]. Patients with cystic echinococcosis displayed significantly increased regulatory T cells and related cytokines (IL-10 and TGF-β) and decreased inflammatory Th17 cells in peripheral blood, indicating the imbalanced Th17/Treg responses may be involved in the immune evasion of the cestode larvae in the parasitized host [59]. Based on its strong immunomodulatory properties, infection with *E. granulosus* has been successfully used to experimentally treat colitis [38] and airway inflammation in mice [43]. The extracts from the *E. granulosus* hydatid-laminated layer also conferred therapeutic effect on dextran sulfate sodium-induced colitis in mice with significantly reduced mucosal damage and inflammatory responses [60]. *Eg*CF also revealed a therapeutic effect on allergic airway inflammation in a mouse model with OVA-induced asthma [61]. Based on the above findings, we explored the therapeutic effect of *Eg*CF on sepsis and sepsis-induced damage to critical organs in this study.

Indeed, after being treated with *Eg*CF, the 72-h survival rate of the mice with CLP-induced sepsis significantly elevated to 40% relative to the septic mice without treatment that all died within this period (Fig. 4). Accordingly, the pathological damage to the liver, lung, and kidney tissue of mice with sepsis was also significantly improved with less inflammatory cell infiltration and reduced pathological damage score (Fig. 5) and improved liver and kidney functions with reduced serological levels of ALT, AST, BUN and Cr (Fig. 6) in *Eg*CF-treated mice. The improved survival rate and reduced pathology in key organs in *Eg*CF-treated mice correlated with the reduced serological levels of inflammatory cytokines TNF-α, INF-γ and increased levels of regulatory cytokines TGF-β and IL-10 (Fig. 7), indicating regulatory immune response is induced that inhibits inflammatory Th1 response. Further investigation identified the transcriptional level of arginase-1 (Arg-1) expressed mostly in M2 macrophages was significantly induced and inducible nitric oxide synthase (iNOS), the marker of M1 macrophages, was significantly inhibited in tissues of liver, lung and kidney of septic mice treated with *Eg*CF, indicating macrophages were polarized from M1 to M2 (Fig. 8) that may contribute to the reduced inflammatory immune responses in *Eg*CF-treated mice. However, we cannot exclude the possibility of the expression of Arg-1 and iNOS by other cells in the tissue even though Arg-1 and iNOS are the major markers of M2 and M1, respectively [62, 63] These results are consistent with the in vitro study with BMDMs incubated with *Eg*CF that induced the M2 macrophage polarization under conditions with LPS (Fig. 2), with reduced levels of inflammatory cytokines TNF-α and INF-γ and increased regulatory cytokines TGF-β, IL-10 (Fig. 3), the similar consequence as septic mice treated with *Eg*CF in vivo. Both in vivo and in vitro results suggest that *Eg*CF can promote macrophage polarization toward the M2 type and inhibit LPS-induced M1 activation under septic conditions. The *Eg*CF-induced M2 polarization may contribute to its therapeutic effect on sepsis and reduce pathology and organ damage caused by excessive inflammation under septic conditions. However, determining the changes of detailed immune cell subpopulations in the sepsis-affected organs would provide more information on the effect of *Eg*CF on the immune responses except for M2 polarization. The passive transfer of in vitro *Eg*CF-stimulated M2 macrophages into septic mice to measure their effect on sepsis in vivo would provide direct evidence of the M2-involved mechanism, and it has been included in our next experiment. The therapeutic effect of *Eg*CF on the sepsis and septic inflammation storm is even stronger than DXM, an anti-inflammatory immunosuppressant commonly used in the treatment of sepsis [64–66]. We also observed that DXM is an immunosuppressant rather than an immunomodulator as *Eg*CF does since the latter has a stronger effect on the induction of TGF-β and Arg-1 expression. It seems that immunomodulation of helminth-derived products plays a more important role in maintaining the immune balance and immune homeostasis than an immunosuppressant in the course of sepsis. TLRs play a key role in maintaining the delicate balance between immune tolerance and activation in the pathogenesis of sepsis [67, 68]. Studies have shown that TLR2 is a major signaling sensor for recognizing microbial infections and is involved in the pathogenesis of sepsis [24]. The TLR-mediated cascade immune response requires the participation of Myd88 as an adaptor or bridge to connect downstream inflammatory signals, which leads to the activation of nuclear transcription factor NF-κB that regulates the expression of inflammatory genes such as TNF-α, IL-1β, and IL-6 y [69]. To investigate if *Eg*CF exerts a protective effect against sepsis by inhibiting the TLR2/MyD88-dependent inflammatory signaling pathway, the protein expression levels of TLR2 and MyD88 in the liver, kidney, and lung tissues of septic mice were examined. The levels of TLR2 and MyD88 were significantly increased in the tissues of septic mice, which was consistent with the high levels of pro-inflammatory factors in the sera of septic mice, indicating that the activation of TLR2/MyD88 signaling pathway in septic mice induced pro-inflammatory mediators. After being treated with *Eg*CF, the expression levels of TLR2 and MyD88 in those key organs (liver, lung, and kidney) of mice with CLP-induced sepsis were significantly reduced (Fig. 9), indicating the anti-inflammatory effect of *Eg*CF on the sepsis is through inhibiting TLR-2 and MyD88 inflammatory signaling pathway. The transcriptome data of septic tissue would provide more details of signal pathways involved in the therapeutic effect of *Eg*CF on sepsis, it will be included in our next study.

## Conclusions

The results of this study demonstrated that the cystic fluid of *E. granulosus* confers a therapeutic effect on sepsis by inhibiting the production of pro-inflammatory cytokines and inducing regulatory cytokines. The anti-inflammatory effect of *Eg*CF is carried out possibly by inducing macrophage polarization from pro-inflammatory M1 to regulatory M2 phenotype to reduce excessive inflammation of sepsis and subsequent multi-organ damage. The role of *Eg*CF in regulating macrophage polarization may be achieved by inhibiting the TLR2/MyD88 signaling pathway. *Eg*CF contains the helminth-derived proteins without infectious larvae (protoscolices), and therefore is a safe reagent to treat inflammatory diseases; however, we are still on the way to identifying the specific components in the cystic fluid that induce immunomodulatory and regulatory functions, as therapeutic targets for autoimmune or inflammatory diseases.

## Data Availability

All datasets presented in this study are included in the article/supplementary material.

## Abbreviations

SIRS: Systemic inflammatory response syndrome
*Eg*CF: *Echinococcus granulosus* cyst fluid
BMDMs: Bone marrow-derived macrophages
CLP: Cecal ligation and puncture
CARS: Compensatory anti-inflammatory response syndrome
TLRs: Toll-like receptors
DXM: Dexamethasone
ALT: Alanine aminotransferase
AST: Aspartate aminotransferase
BUN: Blood urea nitrogen
ELISA: Enzyme-linked immunosorbent assay
IL-6: Interleukin 6
TNF-α: Tumor necrosis factor alpha
TGF-β: Transforming growth factor beta
IL-10: Interleukin 10
